# Shining a light on liver health: advancements in fluorescence-enhanced enzyme biosensors for early disease detection

**DOI:** 10.3389/fbioe.2024.1392857

**Published:** 2024-04-19

**Authors:** Shifeng Liu, Yatong Yin, Shihai Liu, Congxiao Wang, Wenshe Sun, Xiaokun Hu

**Affiliations:** ^1^ Department of the Interventional Medical Center, the Affiliated Hospital of Qingdao University, Qingdao, China; ^2^ Qingdao Maternal and Child Health and Family Planning Service Center, Qingdao, China; ^3^ Medical Research Center, The Affiliated Hospital of Qingdao University, Qingdao, China; ^4^ Qingdao Cancer Institute, Qingdao University, Qingdao, China

**Keywords:** liver disease, enzymes, biosensors, early diagnosis, liver cancer

## Abstract

Early detection of liver diseases holds paramount importance in optimizing treatment outcomes and prognosis, thereby significantly enhancing the likelihood of recovery while mitigating the risk of progression to liver cancer. Liver diseases encompass a spectrum of conditions, each potentially manifesting distinct enzymatic profiles. Monitoring these enzymes *in situ* facilitates timely intervention and therapeutic management. In recent years, the field of biosensor technology has witnessed remarkable advancement, owing to strides in biomedicine and computational sciences. Biosensors have garnered widespread utility across medical and biological domains, spanning the detection of disease biomarkers, drug release tracking, ion imaging, and fluorescence imaging within living organisms. These applications have markedly enhanced imaging resolution and have the potential to refine disease diagnosis accuracy for clinicians. A pivotal aspect in the successful application of this technology lies in the construction of fluorescence probes adept at swiftly and selectively identifying target enzymes by amalgamating liver disease enzymes with fluorescence probe technology. However, research in this niche area remains relatively scarce. Building upon this foundational understanding, the present review delineates the utilization of biosensors in the early diagnosis of liver disease. Serving as a theoretical framework, this review envisages the development of high-performance biosensors tailored for the early detection of liver cancer. Furthermore, it offers insights into the potential of biosensor technology to progress and broaden its practical applications, thus contributing to the advancement of diagnostic methodologies in liver disease management.

## 1 Introduction

The liver serves as the central hub for metabolic activities in the body, orchestrating the conversion of diverse substances, storage of glucose, and secretion of proteins (Asanuma et al., 2015; Asrani et al., 2019; Boursier et al., 2023). As a primary detoxification powerhouse, it possesses the remarkable ability to transform toxic substances into non-toxic or highly soluble compounds, facilitating their safe elimination from the body and safeguarding them against potential harm (Gonçalves et al., 2019; Fabrellas et al., 2020; Fallowfield and Ramachandran, 2021). These pivotal functions render the liver susceptible to pathological changes (Mitchell et al., 2011; Sohrabpour et al., 2012; Schuppan et al., 2018). Liver diseases can be instigated by a variety of factors, including viral infections, medications, organic toxins, ischemia, and autoimmune diseases (Zhang et al., 2020; Sung et al., 2021). Hepatocellular carcinoma (HCC), a prevalent and highly lethal liver disease, currently stands as the sixth most common cancer worldwide and ranks second among malignant tumors associated with cancer-related deaths (Zoubek et al., 2017; Author Anonymous, 2019). Detecting liver cancer in its early stages is of paramount importance. Early diagnosis not only optimizes treatment outcomes but also significantly enhances the survival rate for patients (Wang et al., 2023a). Therefore, the timely identification of liver cancer emerges as a critical factor in elevating the cure rate of this disease (Wang et al., 2023b).

Biomarkers, which are overexpressed in abnormal tissues and diseased cells, hold significant clinical relevance. Extensive clinical trials and practices have underscored the utility of monitoring biomarkers in tissues, serum, and excreta, including reactive oxygen species (ROS), enzymes, miRNAs, and antigens, for disease screening and prognostic evaluation (Li et al., 2015; Liu et al., 2019; Li et al., 2021; Li et al., 2022). Enzymes, in particular, have emerged as highly promising biomarkers, garnering substantial attention owing to their disease specificity and pivotal physiological and clinical roles (Long et al., 2012; Li et al., 2019; Guo et al., 2020; Tang et al., 2020). Notably, the detection of enzyme markers has assumed paramount importance in the strategy for early disease detection (Liu et al., 2018; Yang et al., 2020). For instance, monoamine oxidase B (MAO-B) has been found to be overexpressed in patients with early liver fibrosis, rendering it a promising early marker for liver fibrosis. Similarly, α-L-fucosylase (AFU) exhibits elevated activity in the early stages of liver cancer, preceding lesion formation, thus positioning it as an ideal biomarker for early liver cancer detection (Liu et al., 2018). Additionally, alkaline phosphatase (ALP) enjoys widespread recognition as a biomarker for liver cancer. Therefore, the development of optimal methods for real-time and *in situ* monitoring of enzymes holds the potential to significantly enhance the early and accurate diagnosis of liver diseases (Cheng et al., 2019; Han et al., 2021). To adeptly surveil these biochemical signals within biological entities, the scientific community has engendered an extensive array of organic fluorescent materials (OFMs) tailored for biosensor applications (Wu et al., 2011; Ma et al., 2015; Wang et al., 2016; Zhou et al., 2018; Lim et al., 2021). In juxtaposition with traditional imaging modalities, OFMs proffer a myriad of benefits, encompassing heightened biocompatibility, augmented resolution, and the capacity for real-time monitoring (Alifu et al., 2018; Wang et al., 2020; Geng et al., 2020; Zhang et al., 2022). Specifically, biosensors employing OFMs facilitate extended signal monitoring, thereby enabling meticulous exploration into the dynamics of vital physiological processes (Liu et al., 2017; He et al., 2018). This attribute is particularly advantageous for the longitudinal study of chronic pathologies such as neoplasms and diabetes mellitus (Lu et al., 2014; Qu et al., 2019). The merits of employing OFM-based biosensors also include the diminution of patient discomfort during therapeutic interventions and a notable enhancement in diagnostic precision when contrasted with conventional imaging techniques (Zhang et al., 2016; Zhang et al., 2019).

Building on this foundational substrate, the present scholarly review rigorously delineates the integration of biosensor technologies in the expedient identification of hepatic pathologies. While the existing corpus of literature has perfunctorily engaged with the topic of biosensors, a limited body of work has thoroughly investigated their application in the precocious detection of liver maladies, especially within the ambit of clinical diagnostics. This critique aspires to ameliorate this scholarly void by providing a comprehensive discourse on the myriad sensor architectures and design philosophies implemented in the creation of biosensors specifically designed for this intent. In concert with delineating a diverse array of sensor architectures and design methodologies, this examination delves into varied design strategies for biosensors, reflecting on their imminent clinical applicabilities. Conclusively, this review posits itself as an intellectual edifice for the development of high-precision biosensors aimed at the early identification of liver cancer. It transcends a mere exposition of current trends in biosensor technology to chart out prospective pathways for their enhancement and application in the realm of hepatic disease diagnosis. Additionally, Table 1 provides a snapshot of recent studies on biosensors for reference and further exploration.

**TABLE 1:** The important parameters of enzyme biosensors and some recent studies of biosensors.

Probe	Target	Emission peak (λ_em_) (nm)	Excitation spectra (λ_ex_) (nm)	Ref.
NIR-LAP	LAP	695–770	640	Cheng et al. (2019a)
NIR-ONOO^−^	ONOO^−^	695–770	640	Cheng et al. (2019a)
NIR-NO	cytochrome P450	745	710	Chen et al. (2020)
MitoHCy-NH_2_	monoamine oxidases	770–810	730	Wang et al. (2018a)
DEAN-MA	monoamine oxidase B	456	405	Qin et al. (2019)
probe 1	Carboxylesterases	580	360	Guo et al. (2022)
NIC−1, 2, 3, and 4	carboxylesterase	520	450	Jia et al. (2021)
VPCPP	carboxylesterase	593	443	Qi et al. (2022)
Lyso-CEs	carboxylesterase	575	520	Zhou et al. (2019)
CEMT	Carboxylesterase	570–710	810	Jiang et al. (2019)
L-Pdots	MiRNA-21	425	-	Wang et al. (2021)
N-Pdots	MiRNA-205	672	-	Wang et al. (2021)
DQ-CD@Pdots	GSH	541	450	Sun et al. (2019)
MBTD	lysosomal nitric oxide	565	365	Wang et al. (2018a)
TPBT	double-stranded DNA	537	450	Gao et al. (2019)
HTPQ	alkaline phosphatase	550	410	Liu et al. (2017b)
AFU probe	Alpha-L-fucosidase	605	480	Hou et al. (2017)
MSN@RhB@b-CD@AMPPD	Alkaline phosphatase	580	470	Liu et al. (2018c)
HCA-D	Dipeptidyl peptidase IV	690	640	Guo et al. (2020)

## 2 Liver injury enzyme

Enzymes recognized as highly promising biomarkers, have garnered considerable attention owing to their disease-specific specificity and vital physiological and clinical roles (Nguyen et al., 1991; Okamura et al., 2000; Llovet et al., 2008; Wang et al., 2020; Bhaskar et al., 2023). Notably, the detection of enzyme biomarkers has emerged as a pivotal strategy for the early diagnosis of liver diseases (Lee, 2007; Xiong et al., 2019; Sun et al., 2021; Enbanathan and Iyer, 2022). Liver injury, exemplified by drug-induced liver injury (DILI), represents a significant pathological concern that has emerged as a prominent public health issue (Board and Anders, 2007; Board and Menon, 2013; McDonald and Tipton, 2022). DILI predominantly arises from accidental or intentional medication overdoses, consequently ranking among the leading causes of acute hepatic impairment. Recent advances in biomedical research have underscored the excessive generation of highly reactive oxygen/nitrogen species (ROS/RNS), notably ONOO−, within mitochondria following medication overdose, thereby precipitating severe hepatotoxicity (DiMasi, 2001; Rudin, 2005; Bertolini et al., 2006; Uetrecht, 2010; Amacher et al., 2013). Furthermore, the enzymatic activity of leucine aminopeptidase (LAP) is markedly elevated during hepatic dysfunction compared to physiological states (Aithal et al., 2011; Wang and Zhou, 2013; Atallah et al., 2021). Notably, both ONOO− and LAP have been substantiated as early diagnostic biomarkers for drug-induced liver diseases by numerous investigators (Pacher et al., 2007; Howell et al., 2018).

**FIGURE 1:**
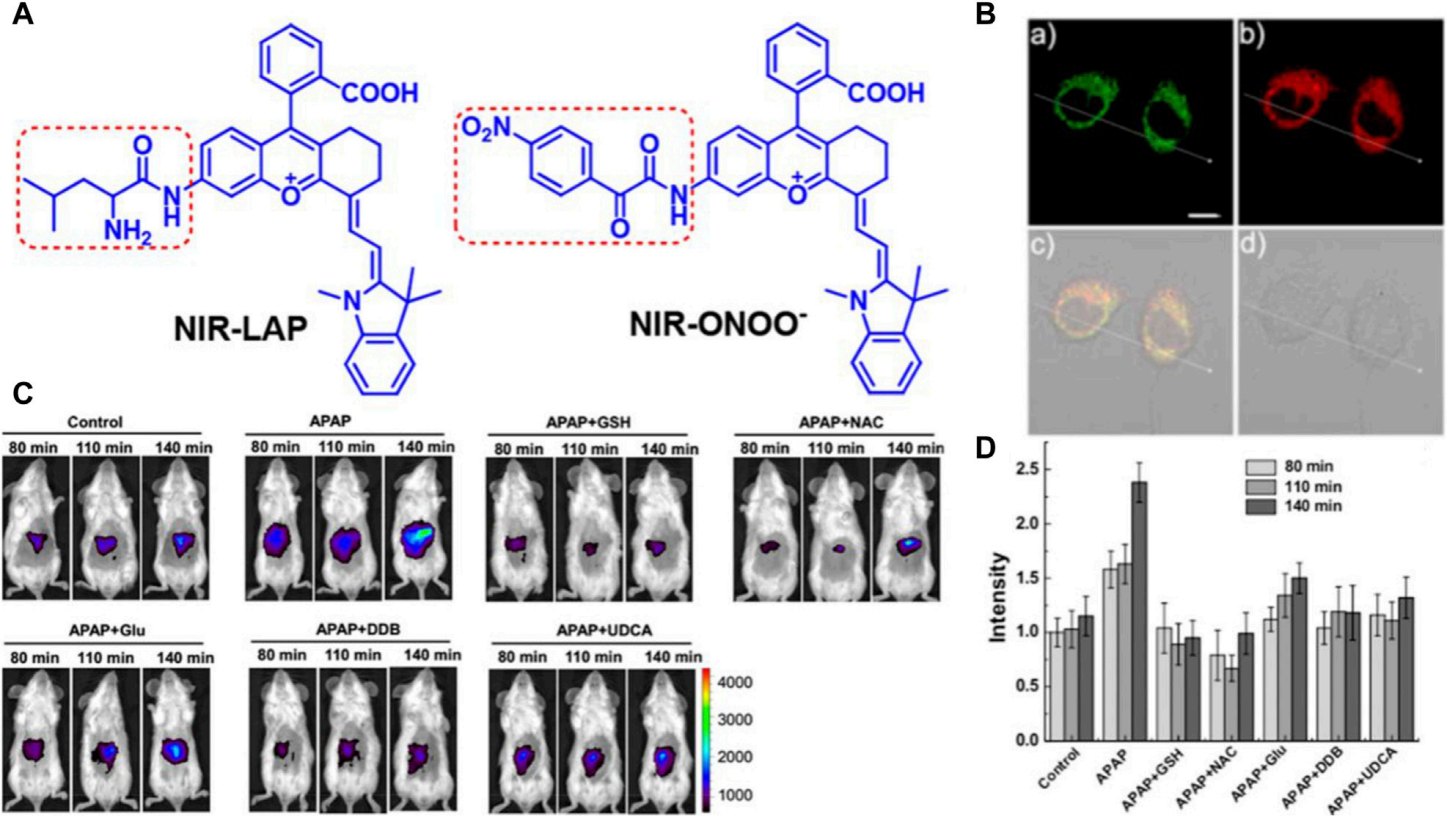
**(A)** The chemical structure of compounds NIR-LAP and NIR-ONOO−. Adapted with permission from (Cheng et al., 2019) **(B)** The co-localization of NIR-LAP in HepG2 cells (Pearson’s correlation coefficient (r) = 0.82). a) Green channel: Mito-Tracker Green (1 μM) stain (λex = 488 nm, λem = 500–550 nm); b**)** red channel: NIR-LAP (5 μM) stain (λex = 640 nm, λem = 663–738 nm); c) yellow: merged signal. d) reference. Scale bar: 10 μm. Adapted with permission from (Cheng et al., 2019) **(C)** representative images of BALB/c mice receiving saline (control), APAP (300 mg kg^−1^, intraperitoneally) alone, or with GSH (200 mg kg^−1^, intravenously), NAC (150 mg kg^−1^, intravenously), Glu (200 mg kg^−1^, intravenously), DDB (200 mg kg^−1^, intraperitoneally), UDCA (20 mg kg^−1^, intraperitoneally), followed by NIR-LAP (60 μL, 100 μM, intravenously) (λex = 640 nm, λem = 695–770 nm). Adapted with permission from (Cheng et al., 2019) **(D)** Relative fluorescence intensity in **(C)**. Data are expressed as mean ± SD of three experiments. Adapted with permission from (Cheng et al., 2019).

Based on the above, Xiaobing Zhang’s team first time to showcase the drug-induced hepatotoxicity pathways by use of a small-molecule fluorescent probe (Cheng et al., 2017), and further innovatively designed a novel near-infrared fluorescent dye with adjustable optical signals, employing it as the fluorescent precursor to synthesize two highly precise near-infrared fluorescent probes, namely, NIR-LAP and NIR-ONOO−, for detecting liver toxicity markers leucine aminopeptidase (LAP) and peroxynitrite (ONOO−), respectively (see Figure 1A) (Cheng et al., 2019). These probes exhibit no inherent fluorescence; however, upon interaction with LAP or ONOO−, they produce the product NIR-NH_2_, consequently restoring fluorescence. This unique feature enables the sensitive and accurate detection of LAP and ONOO−. The efficacy of these probes was demonstrated through the detection of LAP and ONOO− within cell mitochondria (see Figure 1B). Furthermore, the probes were utilized to visualize LAP and ONOO− fluctuations in mice with drug-induced liver injury (see Figure 1C). Leveraging the high sensitivity and specificity of the probes, an accurate evaluation of drug-induced liver toxicity became feasible. Lastly, the probes were successfully employed to assess the therapeutic effects of six liver-protective drugs on acetaminophen-induced liver toxicity (see Figure 1D). The high-fidelity NIR dye platform with an optically tunable group might provide an efficient method for the development of future probes applied in the pathological environment. This optically tunable, high-fidelity near-infrared dye not only offers a convenient and effective platform but also holds great promise for the design and development of pathological environmental probes. Nevertheless, while the NIR-LAP and NIR-ONOO− demonstrate considerable promise in monitoring the hepatic health status during pharmacotherapy, the evaluative metrics employed to gauge liver health are notably deficient in cogency. This deficiency represents a significant quandary within the domain of biosensor research, necessitating the incorporation of more rigorous, professional medical evaluative methodologies pertaining to hepatic function, such as the Histological Activity Index. This gap underscores an imperative need for the advancement of diagnostic criteria that can more effectively interface with the nuanced capabilities of biosensor technology in the context of liver health assessment.

**FIGURE 2:**
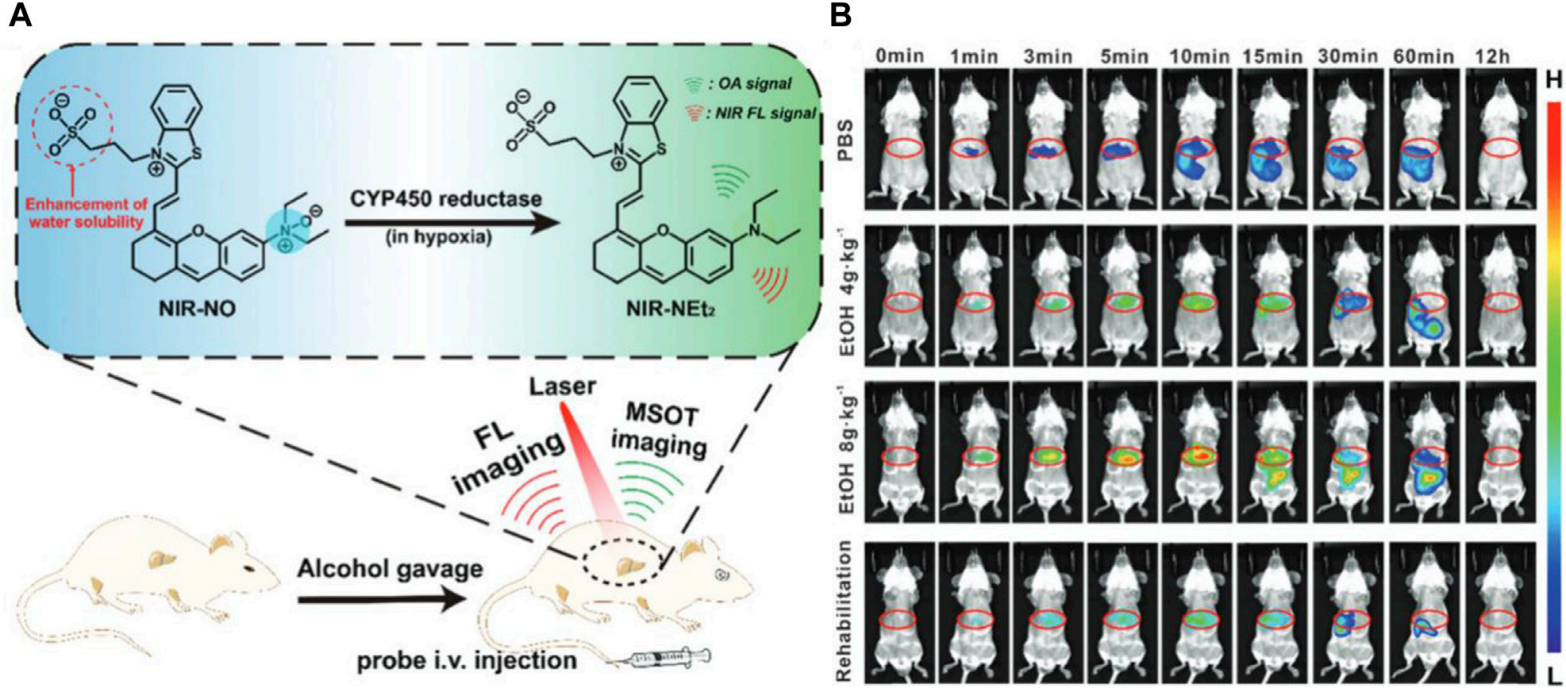
**(A)** Schematic display of the probe’s response to the over-expressed CYP450 reductase in the hepatic region and fluorescent and MSOT imaging of alcohol-induced liver injury in a mouse model. Adapted with permission from (Chen et al., 2020) **(B)** Time course for fluorescence imaging *in vivo*. The mice were pretreated with PBS, 4 g kg^−1^ EtOH, 8 g kg^−1^ EtOH, or 8 g kg^−1^ EtOH +200 mg kg^−1^ methadone once a day for three consecutive days, followed by tail intravenous injection of 2.7 mg kg^−1^ NIR-NO in PBS. Adapted with permission from (Chen et al., 2020).

Alcoholic liver diseases (ALDs) stand as the predominant cause of advanced liver conditions, representing a significant contributor to liver-related mortality on a global scale (Nath and Szabo, 2012). Alcohol-induced liver injury marks the early stages of ALDs, encompassing hepatic lesions, fatty liver, steatohepatitis, progressive fibrosis, cirrhosis, and, in severe cases, liver failure (Louvet and Mathurin, 2015). Detecting alcohol-induced liver injury holds paramount importance for devising optimal rehabilitation programs and effective treatment strategies. Addressing this, the research group led by Wu Shuizhu has introduced a water-soluble enzyme-activated fluorescent probe, named NIR-NO, designed for photoacoustic/fluorescence dual-mode imaging detection of alcohol-induced liver injury (Chen et al., 2020). In the chemical structure of the probe, the benzothiazole-anthracene chromophore serves as the signal reporter, while the N-oxide moiety, endowed with fluorescence-inhibiting ability, acts as a responsive group for cytochrome P450 (CYP) reductase (see Figure 2A). Upon interaction with CYP450 reductase, the substrate undergoes deoxygenation and reduction, resulting in the release of fluorophores and the generation of robust near-infrared fluorescence. Simultaneously, the probe’s absorption undergoes a redshift to the near-infrared region, yielding intense photoacoustic signals. Leveraging the dual signals of fluorescence and photoacoustic, the probe effectively detected mice with alcoholic liver injury (see Figure 2B). Moreover, the fluorescence intensity exhibited a noticeable increase with escalating ethanol (ETOH) concentrations, offering a means to indicate the stage of ALDs and aid in clinical diagnosis. This probe not only emerges as a promising tool for studying the physiological and pathological processes associated with ALDs but also holds potential significance when combined with sensors to estimate enzyme activity changes. Currently, a multitude of fluorescence imaging modalities are available for the *in situ* examination of enzymatic markers associated with hepatic carcinoma. However, persistent challenges call for continued refinement, especially in the innovation of supplementary near-infrared (NIR-II) and photoacoustic dual-mode fluorescent probes. The evolution of these technological modalities harbors the potential to markedly amplify both sensitivity and the signal-to-noise ratio, thus paving the way for more precise and effective diagnostic capabilities in the realm of liver cancer detection.

## 3 Liver fibrosis enzyme

Liver fibrosis denotes the aberrant proliferation of connective tissue within the liver triggered by diverse pathogenic factors. Any form of liver injury initiates a cascade culminating in liver fibrosis during the organ’s reparative phase. Prolonged exposure to injurious stimuli can perpetuate the fibrotic process, ultimately leading to cirrhosis. Monoamine oxidase (MAO, EC 1.4.3.4) is a flavin-dependent enzyme that mediates the oxidation of amine substrates to corresponding imines (Westlund et al., 1985). Widely distributed in tissues, MAO exhibits the highest expression levels in the liver (Denney et al., 1982). MAO exists as two isoenzymes, namely, monoamine oxidase A (MAO-A) and monoamine oxidase B (MAO-B), with the latter implicated in collagen fiber formation and closely associated with liver fibrosis (Kim et al., 2018). Numerous investigations have identified elevated MAO-B levels in the serum of patients with early liver fibrosis, rendering it an ideal biomarker for early fibrosis diagnosis (Walker and Edmondson, 1994; Park et al., 2019). Hence, elucidating MAO-B activity levels in cells and *in vivo* holds promise for facilitating early liver fibrosis diagnosis and unveiling its pivotal role in fibrotic pathogenesis.

**FIGURE 3:**
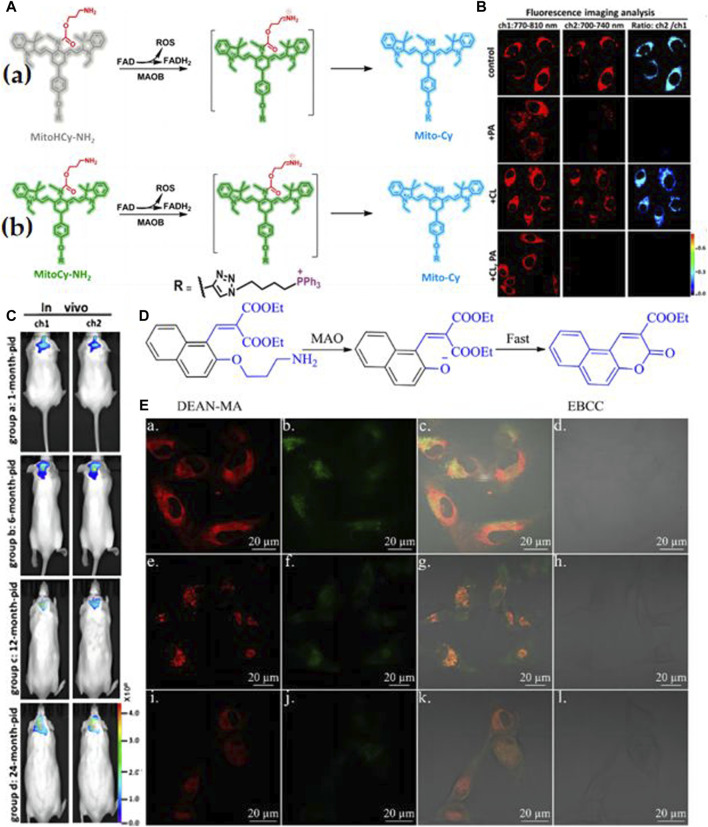
**(A)** a) Synergistic detection mechanism of MitoHCy-NH_2_ toward MAO-B and reactive oxygen species (ROS); b) the detection mechanism of MitoCy-NH_2_ toward MAO-B. Adapted with permission from (Wang et al., 2018). **(B)** Ratiometric fluorescence images for the selectivity of MitoCy-NH_2_ in SMMC7721 cells. Two fluorescence collection windows were used: channel 1, 750–800 nm (λex = 730 nm); channel 2,700–740 nm (λex = 650 nm). All the cells were washed three times with fresh DMEM before imaging. (a) Group a: incubated with 5 μM MitoCy-NH 2 for 100 min as control. Group b: incubated with 10 μM PA for 120 min, and then treated as described in group a. Group c: incubated with 10 μM CL for 120 min, and then treated as described in group a. Group d: incubated with 10 μM PA and 10 μM CL for 120 min, and then treated as described in group a. Adapted with permission from (Wang et al., 2018). **(C)** Imaging of MAO-B levels in mice brains with different ages. Fluorescence collection windows were the following: channel 1, 760–840 nm, λex = 730 nm; channel 2, 700–800 nm, λex = 650 nm. All the BALB/c mice were incubated with MitoCy-NH_2_ (100 μM, 50 μL in 1:99 DMSO/saline, v/v) for 30 min with intracranial injection. (a) *In vivo* imaging of mice in groups a−d; the ages of the mice were 1, 6, 12, and 24 months old, respectively. Adapted with permission from (Wang et al., 2018). **(D)** The “covalent assembly” strategy is applied in this work. Adapted with permission from (Qin et al., 2019). **(E)** The fluorescence image of U87 cells incubated with DEAN-MA and mito-tracker (a, b, c, d), lyso-tracker (e, f, g, h), and ER-tracker (i, j, k, l). a, e, i for the red channel of tracker; b, f, j for the blue channel of DEAN-MA; c, g, k for overlay; d, h, l for bright. H. Qin et al./Chinese Chemical Letters 30 (2019) 71–74 73. Adapted with permission from (Qin et al., 2019).

Rui Wang and colleagues have introduced two innovative near-infrared (NIR) fluorescent probes, MitoCy-NH_2_ and MitoHCy-NH_2_, designed for synergistic imaging of MAO-B and analysis of its role in oxidative stress within cellular and mouse aging models (Wang et al., 2018). These probes comprise three components: cyanine as the fluorescent moiety, propionamide as the recognition element, and triphenylphosphine cation as the mitochondrial targeting unit (Figure 3A). MitoCy-NH2 offers near-infrared fluorescence ratio signals (Figure 3B) and has been effectively utilized for the selective detection of MAO-B in H_2_O_2_-induced cellular and mouse aging models (Figure 3C). In a parallel endeavor, Yu Xiaoqi’s research team developed a 3-aminopropyl salicylaldehyde derivative probe, DEAN-MA, for MAO-B detection (Qin et al., 2019). Upon oxidation and enzymatic cleavage, the 3-aminopropyl groups are exposed to hydroxyl groups, triggering cyclization to generate coumarin with robust fluorescence (Figure 3D). Utilizing this probe, the authors successfully observed significantly higher MAO-B levels in human astrocytes (U87) compared to human liver cells (HL-7702) (Figure 3E). These observations emphatically highlight the considerable worth and prospective utility of probes within the sphere of Monoamine Oxidase B (MAO-B) research. Nevertheless, there is a cogent necessity for further enhancements to augment the stability of MAO-B probes. Such advancements are imperative to guarantee their persistent activity over extended durations within intricate biological matrices, thereby mitigating the likelihood of erroneous positive or negative results.

## 4 Liver cancer enzyme

Liver cancer, a formidable and often lethal malignancy, presents a challenging landscape with a low survival rate for affected individuals. Early detection and intervention stand out as pivotal strategies to enhance patient survival rates. Hepatocellular carcinoma, characterized by considerable heterogeneity, transcends being a mere mass of proliferating cancer cells; it is a complex tissue comprising various cell types (Rodriguez-Rios et al., 2022). Consequently, accurately identifying nascent nodes of liver cancer through physical means proves challenging. In contrast, a more practical approach involves precise identification of its specific molecular characteristics for early diagnosis, particularly focusing on its distinctive biomarkers (Wu et al., 2022). The ideal biomarker for early liver cancer should exhibit high sensitivity and specificity, enabling noninvasive detection with reproducible results. Presently, several new biomarkers, such as AFP, lectin-reactive alpha-fetoprotein, des-gamma-carboxyprothrombin, and Golgi protein 73, have been identified as effective indicators of liver cancer. Additionally, proteins with heightened expression in the liver emerge as potential candidate biomarkers, including carboxylesterase 1, hemoglobin alpha, alcohol dehydrogenase 1b, alcohol dehydrogenase 4, and glucose regulatory protein 78. These advancements in biomarker identification contribute to the ongoing pursuit of more accurate and reliable methods for early liver cancer diagnosis.

Carboxylesterase (CE), a prominent endoplasmic reticulum protein highly expressed in the liver, possesses the ability to be secreted outside the cell, disrupting calcium homeostasis within the endoplasmic reticulum lumen (Liu et al., 2018). This disruption can lead to endoplasmic reticulum dysfunction, inducing energy and endogenous metabolic impairments, oxidative stress, and ultimately resulting in liver cell injury and carcinogenesis (Li et al., 2019). Consequently, CE emerges as a potential diagnostic and analytical indicator for liver cancer. The design of CE fluorescent probes predominantly adopts the “inhibitor-derived” strategy, a classic approach in enzyme probe design (Fan et al., 2021; Wu et al., 2021). In this strategy, the active part or entire structure of a classic enzyme inhibitor is integrated and coupled with a fluorescent chromophore to regulate fluorescence release. The rational design of a practical probe with excellent specificity for specific enzymes and improved optical properties remains a significant challenge.

**FIGURE 4:**
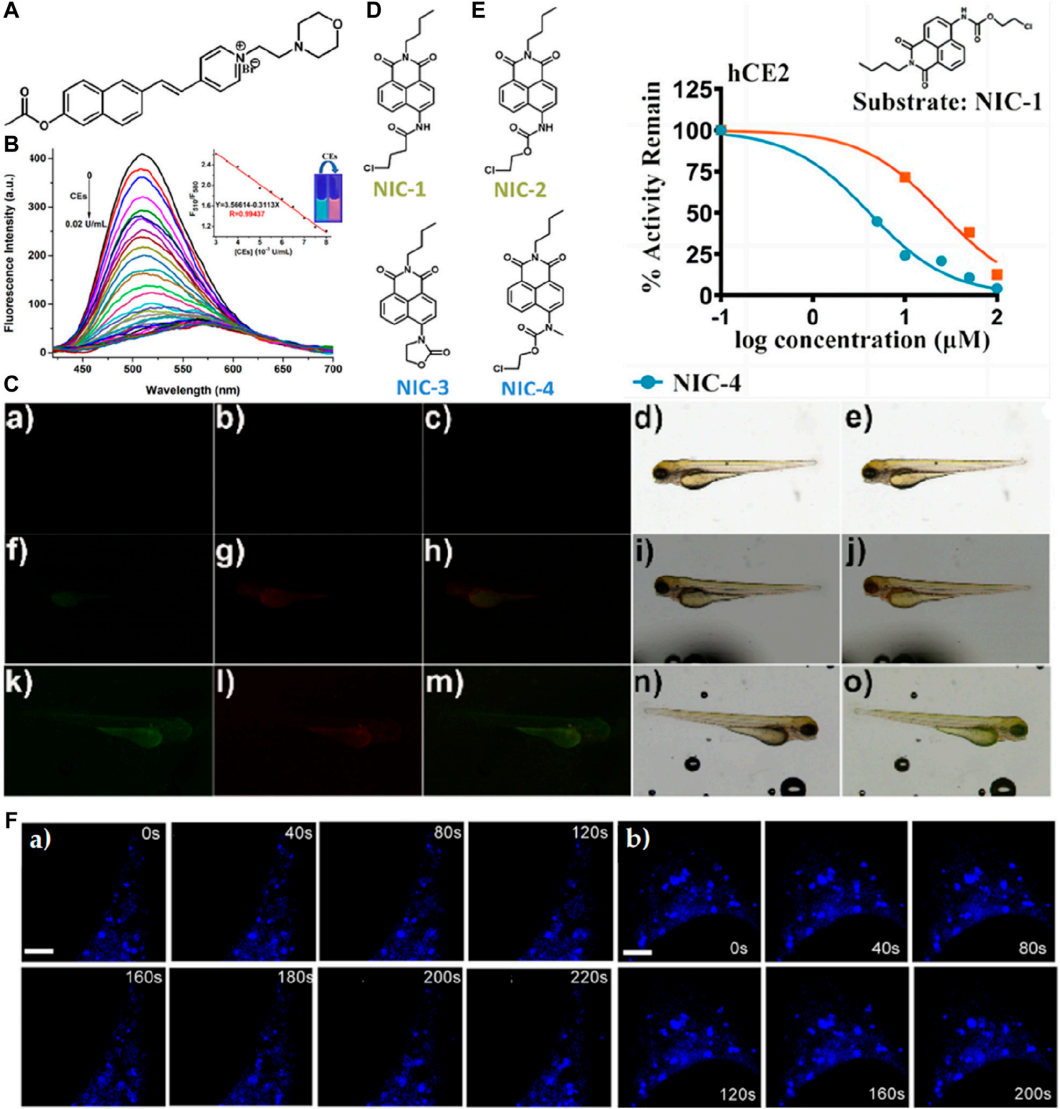
**(A)** The chemical structure of probe 1. **(B)** Fluorescence spectra of 10 μM probe 1 upon the addition of CEs (0–0.02 U/mL.) in PBS buffer solution (10 mM, pH 7.4). The insert shows the fluorescence intensity from 510 nm to 580 nm as a function of CE concentration with a linear relationship. The excitation wavelength was 360 nm. Excitation and emission slit widths are 5 nm each. Adapted with permission from (Guo et al., 2022). **(C)** Fluorescence images of the sensor in living zebrafish: (a, f, k) green channel; (b, g, l) red channel; (c, h, m) the merged images of (a, f, k) and (b, g, l); (d, i, n) bright field; (e, j, o) the merged images of (c, h, m) and (d, i, n). (a–e) in the absence of the probe; (f–j) incubated with probe 1 (10 μM) for 30 min; (k–o) pretreated with BNPP (100 μM) for 30 min before incubating with probe 1 for another 30 min; Adapted with permission from (Guo et al., 2022). **(D)** Structurally diversified carbamates designed and developed serve as inhibitors or substrates of hCEs in this study. Adapted with permission from (Jia et al., 2021). **(E)** Inhibition properties of NIC series. NIC−1 hydrolysis in hCE2. Inhibition types for NIC-3/4 were defined by the Lineweaver−Burk analysis. Adapted with permission from (Jia et al., 2021). **(F)** Time-lapse SIM images of different areas of hCE1 movement, representatively. For the time-lapse images, the time interval between each SIM image was set to 20 s with a course of 4 min. Scalebar: 5 μm in Figure c) and 4 μm in d). Adapted with permission from (Jia et al., 2021).

Guo et al. devised a water-soluble semi-cyanine fluorescent probe named 1 aimed at detecting cholinesterase (CE) activity through the incorporation of acetate bond recognition sites (Figure 4A) (Guo et al., 2022). *In vitro* fluorescence assays revealed that upon the addition of CE, the emission of the probe’s main peak at 510 nm was partially quenched and red-shifted to 580 nm. Notably, the probe exhibited high sensitivity to CE activity (with a detection limit of 0.98 × 10^−6 ^U/mL) and demonstrated rapid response kinetics (<10 min) (Figure 4B). Moreover, owing to the presence of morpholine groups, the probe exhibited the capability to target lysosomes and monitor CE activity within these organelles. Successful application of the probe was demonstrated in the detection and imaging of CE activity in zebrafish (Figure 4C). In a separate study in 2021, the Han research group introduced a series of CE fluorescent probes utilizing carbamate as a foundational moiety named NIC−1, NIC-2, NIC-3, and NIC-4 (Figure 4D), subsequently employing them for super-resolution microscopy imaging of CE activity in live cells (Jia et al., 2021). Results underscored the efficacy of the NIC-4 as a CE inhibitor, whereby methyl substitution of nitrogen in the carbamate moiety effectively impeded the proton transfer process, thereby hindering nucleophilic attacks necessary for CE hydrolysis (Figure 4E). Intriguingly, cellular imaging elucidated the probe’s capability to dynamically track the flux of CE in live cells in real-time (Figure 4F).

**FIGURE 5:**
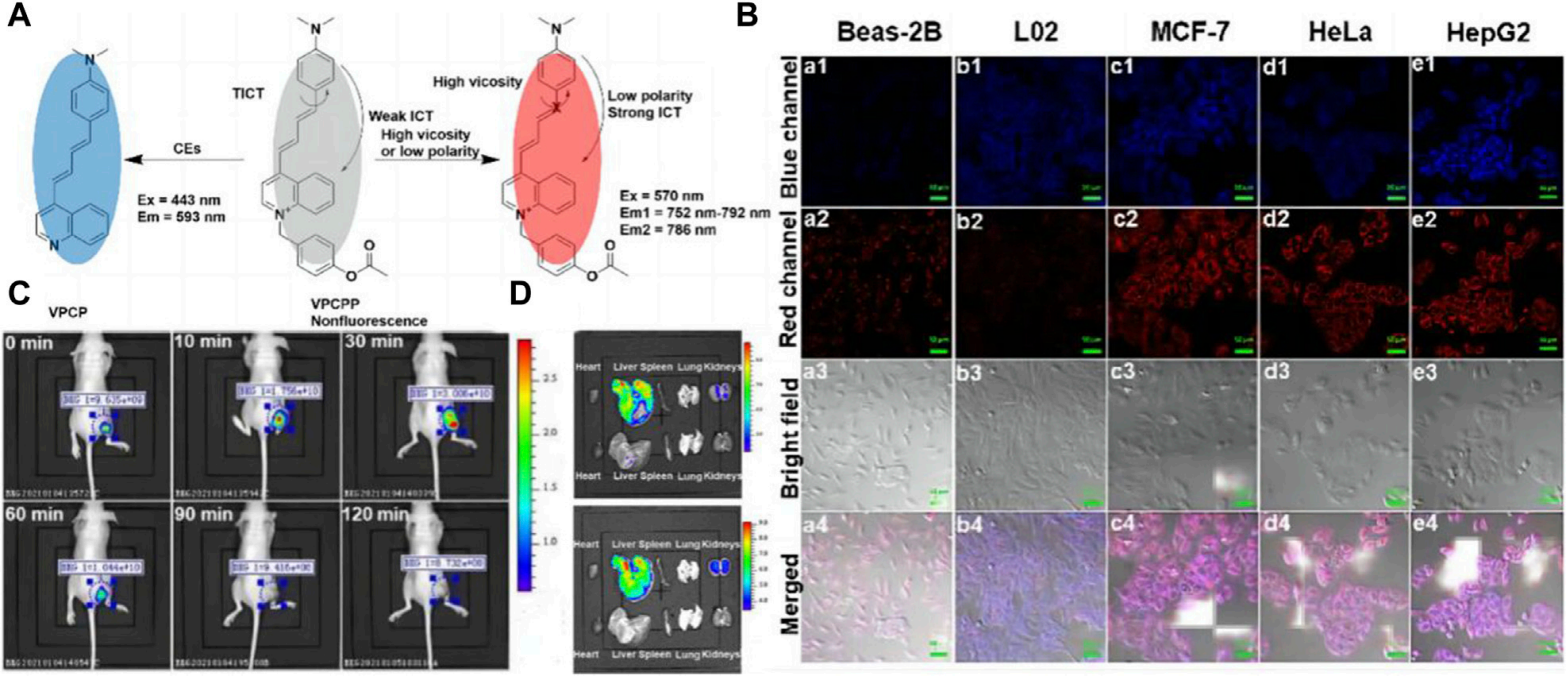
**(A)** OR Logic Gate-Based Fluorescent Probe VPCPP for Monitoring Local Microviscosity, Micropolarity, and CEs. Adapted with permission from (Qi et al., 2022) **(B)** Confocal fluorescence images for detecting local microviscosity, micropolarity, and CEs in various cell lines incubated with VPCPP (5 μM) for 1 h (a1−a4): Beas-2B cells. (b1−b4): L02 cells. (c1−c4): MCF-7 cells. (d1−d4): HeLa cells. (e1−e4): HepG2 cells. Adapted with permission from (Qi et al., 2022) **(C)** *In vivo* imaging of the 4T1 cells tumor-bearing mice after intratumoral injection of VPCPP (10 μM, 50 μL). Adapted with permission from (Qi et al., 2022) **(D)** *Ex vivo* imaging of main organs isolated from mice. Upline: the mouse intravenous injected with VPCPP (200 μM, 100 μL). Down the line: the mouse was intravenously injected with saline (0.9%, 100 μL). λex = 430 nm; λem = 575–650 nm and λex = 570 nm; λem = 695–770 nm. Adapted with permission from (Qi et al., 2022).

In 2022, Zhu’s team achieved a notable milestone by developing a near-infrared fluorescent probe named VPCPP (see Figure 5A) (Qi et al., 2022). This probe enables simultaneous monitoring of local microviscosity, micro-polarization, and CE in living cells through both blue and red channels. VPCPP demonstrated its ability to distinguish between cancer cells and normal cells, including BEAS-2B (human bronchial epithelium cell line), L02 (human normal liver cell line), HeLa (human cervical cancer cell line), MCF-7 (human breast adenocarcinoma cell line), and HepG2 (human liver hepatocellular carcinoma cell line) (see Figure 5B). The authors induced CE overexpression by employing aristolochic acid, a carcinogen capable of upregulating CE expression, and successfully imaged liver tumor-bearing mice using both two-channel and single-channel imaging (see Figures 5C, D). This breakthrough underscores the potential of VPCPP as a powerful tool for studying liver cancer and paves the way for future advancements in diagnostic and therapeutic strategies. Nonetheless, an issue that necessitates rectification persists within the domain of biosensor design; notably, the chemical architecture of many biosensors frequently encompasses a plethora of unstable structures, including ether bonds. Such configurations markedly compromise the stability of the sensors, particularly in scenarios necessitating long-term tracking. Furthermore, the incorporation of a fully conjugated structure within biosensors emerges as a beneficial strategy for bolstering their stability. This approach not only addresses the inherent fragility associated with certain chemical linkages but also paves the way for enhanced performance and reliability of biosensors in prolonged investigative applications.

**FIGURE 6:**
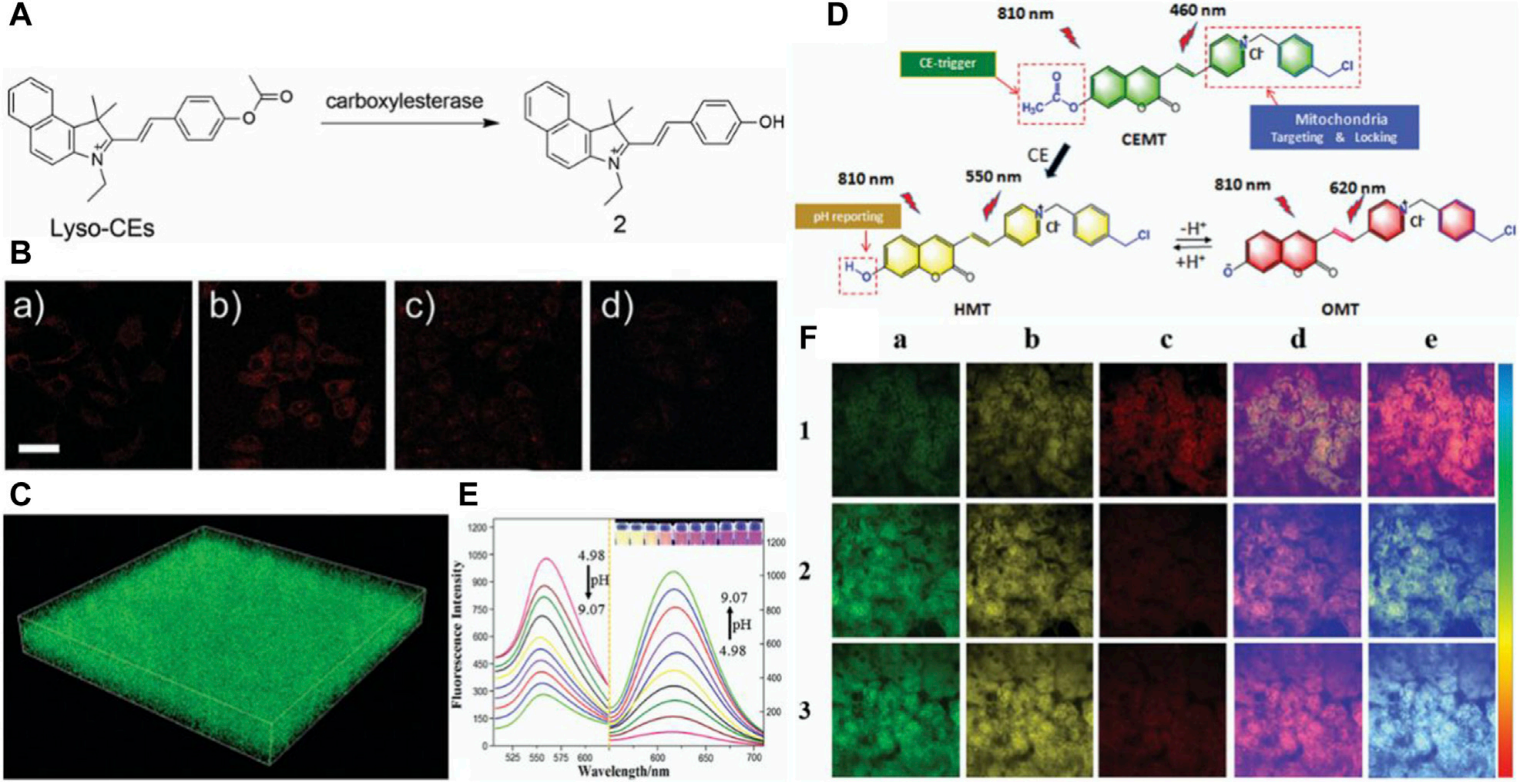
**(A)** The chemical structure of the probe Lyso-CEs and its reaction with CEs. Adapted with permission from (Zhou et al., 2019) **(B)** Confocal fluorescence images of cells. (a) A549 cells were incubated with 10 mM Lyso-CEs; (b) HepG2 cells were incubated with 10 mM Lyso-CEs; (c) HepG2 cells were pretreated with 50 mM AEBSF for 30 min, and then incubated with 10 mM Lyso-CEs; (d) HepG2 cells were pretreated with 100 mM AEBSF for 30 min, and then incubated with 10 mM Lyso-CEs. The bottom row is the corresponding differential interference contrast (DIC) images. Scale bar, 40 μm. Adapted with permission from (Zhou et al., 2019) **(C)** 3D fluorescence images in mice liver slices incubated with Lyso-CEs (10 mM) for 30 min at 37°C from confocal Z-scan imaging sections at a depth of 0–60 μm. lex = 514 nm, lem = 520–650 nm, scale bar: 100 μm. Adapted with permission from (Zhou et al., 2019) **(D)** Design and sensing mechanism of a ratiometric two-photon mitochondria-locked probe CEMT toward carboxylesterase (CE) activity and pH. Adapted with permission from (Jiang et al., 2019) **(E)** Fluorescence spectra change in the presence of 40 mM B–R buffer solution at various pH (4.98, 5.31, 5.74, 6.01, 6.50, 7.08, 7.42, 8.31, 8.59, 9.07) (lex = 410 nm and 530 nm). Adapted with permission from (Jiang et al., 2019) **(F)** Two-photon fluorescence images for the liver of mice with cardiac perfusion: (1) CEMT (20 mM) for 10 min (2) Pretreated with vinyl acetate (1 mM) before with CEMT (20 mM). (3) Pretreated with benorilate (1 mM) before with CEMT (20 mM) for 10 min. Images were captured using 810 nm for two-photon excitation. Emission window: (a) green channel (400,500 nm). (b) Yellow channel (500–570 nm). (c) Red channel (570–710 nm). (d) Ratio imaging: I_yellow_/I_green_. (e) Ratio imaging: Ired/Iyellow. Scale bar: 20 mm. Adapted with permission from (Jiang et al., 2019).

Ma’s research group devised and synthesized a novel red “turn-on” lysosome-targeted fluorescent probe, termed Lyso-CEs, by combining hemocyanins as a quenching group with an acetyl group as a recognition group (see Figure 6A) (Zhou et al., 2019). Lyso-CEs undergo facile hydrolysis, liberating the fluorophore hydroxy hemicyanine and emitting an off-on spectroscopic signal upon encountering CE. This probe exhibits high selectivity and sensitivity in the fluorescence detection and imaging of CE in live cells (see Figure 6B), serum, and tissues (see Figure 6C). In a parallel endeavor, Jiang et al. synthesized a two-photon ratiometric fluorescent probe named CEMT (see Figure 6D), leveraging coumarin as the fluorescent parent nucleus to target mitochondria. CEMT was designed for detecting and imaging CE activity and pH in mitochondria (Jiang et al., 2019). The combined influence of CE and pH induces noticeable changes in the fluorescence intensity of the probe (see Figure 6E), holding promise for monitoring drug delivery processes. *In vitro* cell imaging substantiates the exceptional targeting ability of CEMT, which tightly homes in on mitochondria. To monitor drug metabolism in the liver, drug administration was performed in mice via heart perfusion with vinyl acetate or benorilate, as depicted in Figure 6F. The results highlight the potential application of CEMT in CE-mediated acidification during anticancer drug administration. This comprehensive approach not only underscores the probe’s significance in biosensors but also emphasizes the critical role it plays in studying drug metabolism. To this juncture, notwithstanding the significant advancements realized in the fabrication of CE biosensors, there persists an imperative for the augmentation of their anti-interference faculties. This exigency emanates from the inherent vulnerability of CE sensors to potential reciprocal interference instigated by other esterases, most prominently AChE and BChE. These enzymes, sharing analogous recognition sites with CE, pose a substantial challenge to the specificity and accuracy of CE sensor readings, thereby underscoring the critical need for technological refinements to mitigate such interference (Zhu et al., 2015; Zhou and Ma, 2016; Zheng et al., 2017; Gao et al., 2020; Chen et al., 2021; Zhang et al., 2023).

## 5 Conclusion

Liver disease stands as a prevalent and highly detrimental condition with a propensity for progressing into liver cancer. Enhancing patient survival rates hinges significantly on the early detection and intervention of liver disease. Among the myriad of detection methods, fluorescence probe technology has garnered attention for its *in situ* applicability, multi-level analysis, and high sensitivity. Scientists have diligently developed various fluorescent probes tailored for different liver disease enzymes. Extensive clinical trials and practical applications have underscored the effectiveness of monitoring enzymes. This approach proves instrumental in disease screening and prognostic assessment. The diverse applications of fluorescence probe technology have markedly elevated imaging resolution, equipping healthcare professionals with more precise tools for disease diagnosis. This, in turn, facilitates real-time and accurate detection, contributing significantly to the meaningful early diagnosis of liver disease.

**FIGURE 7:**
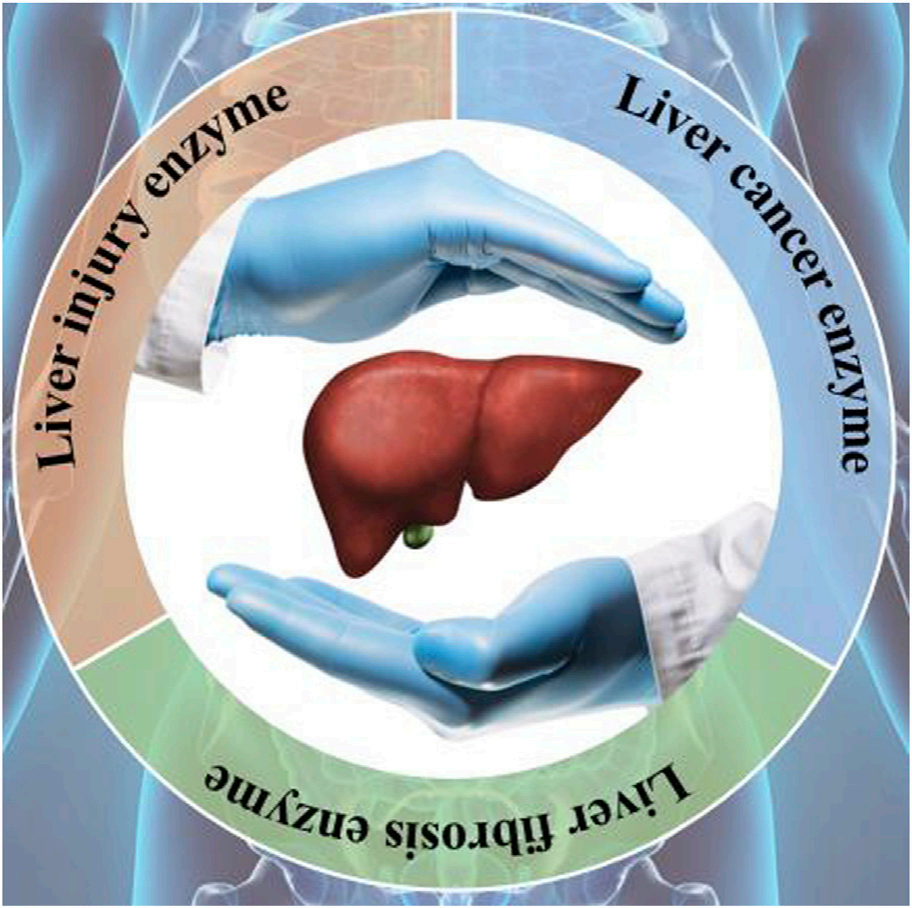
Enzyme biosensors for various liver diseases.

We categorically assert that enzyme biosensors designed for the diagnosis of liver disease hold considerable promise for clinical application (Figure 7). Yet, there remains a substantive gap between their current state and practical deployment in a clinical setting. From a clinical vantage point, it is imperative, firstly, to broaden the scope of toxicological investigations to ascertain that such biosensors do not adversely affect patient health—a fundamental criterion for their use. This includes ensuring that the biosensors do not detrimentally impact not only the target organ but also other physiological systems. Secondly, there is a pressing need to incorporate more sophisticated medical indicators into biosensor research, a measure that would facilitate the seamless integration of these devices within the medical field and catalyze their advancement. Such an approach would not only enhance the relevance of biosensors to clinical diagnostics but also ensure that their development aligns with the overarching objectives of medical science, thereby fostering a more holistic approach to patient care and treatment. Besides these challenges above, to further propel the development of high-performance enzyme biosensors and facilitate their commercialization, several crucial challenges pertaining to biomarker fluorescent probes need to be addressed: i) Evaluating the potential impact of sensors on the physiological activities of organisms is paramount. ii) Overcoming the limitation posed by the water solubility of biomarker fluorescent probes is crucial for expanding their applications. iii) Ensuring the metabolism period of biomarkers aligns with the intended tracking period is essential. iv) Designing high-performance biomarker biosensors necessitates the integration of multiple advantages into a single molecule, such as strong fluorescence in the NIR or NIR-II region, excellent stability, and biocompatibility without toxicity. v) Exploring and developing more applications for biomarker fluorescent probes will further enhance their versatility and utility. Addressing these challenges will undoubtedly contribute to the maturation of enzyme probe technology and its broader adoption in various biomedical applications.
